# A New Look at the Daily Cycle of Trade Wind Cumuli

**DOI:** 10.1029/2019MS001746

**Published:** 2019-10-16

**Authors:** Jessica Vial, Raphaela Vogel, Sandrine Bony, Bjorn Stevens, David M. Winker, Xia Cai, Cathy Hohenegger, Ann Kristin Naumann, Hélène Brogniez

**Affiliations:** ^1^ Max‐Planck‐Institut für Meteorologie Hamburg Germany; ^2^ Laboratoire de Météorologie Dynamique Universite Pierre et Marie Curie (UPMC) Paris France; ^3^ NASA Langley Research Center Hampton VA USA; ^4^ Science Systems and Applications, Inc. Hampton VA USA; ^5^ Laboratoire Atmosphères, Milieux Observations Spatiales Paris France

**Keywords:** daily cycle, diurnal time scale, trade wind cumuli, shallow clouds, observations, high‐resolution models

## Abstract

A description of the daily cycle of oceanic shallow cumulus for undisturbed boreal winter conditions in the North Atlantic trades is presented. Modern investigation tools are used, including storm‐resolving and large‐eddy simulations, runover large domains in realistic configurations, and observations from in situ measurements and satellite‐based retrievals. Models and observations clearly show pronounced diurnal variations in cloudiness, both near cloud base and below the trade inversion. The daily cycle reflects the evolution of two cloud populations: (i) a population of nonprecipitating small cumuli with weak vertical extent, which grows during the day and maximizes around sunset, and (ii) a population o deeper precipitating clouds with a stratiform cloud layer below the trade inversion, which grows during the night and maximizes just before sunrise. Previous studies have reported that cloudiness near cloud base undergoes weak variations on time scales longer than a day. However, here we find that it can vary strongly at the diurnal time scale. This daily cycle could serve as a critical test of the models' representation of the physical processes controlling cloudiness near cloud base, which is thought to be key for the determination of the Earth's climate response to warming.

## Key Points

- Trade wind cumuli exhibit a pronounced daily cycle, with an evening maximum near cloud base and a nighttime/dawn maximum near the inversion
- Cloud‐base cloudiness during undisturbed periods can be much more variable at the diurnal than at longer time scales
- The representation of the daily cycle of inversion clouds is particularly sensitive to resolution

## 1 Introduction

There is increasing evidence that oceanic fair‐weather cumulus clouds, such as those prevailing in the trade wind regions, play an important role in the climate system. They are ubiquitous over vast areas of the tropical and subtropical oceans and as such have a strong influence on the planetary albedo (Medeiros et al., 2008). Predicting how they might change in response to global warming, and how those changes impact the Earth's radiative budget—and by this the global cloud feedback and climate sensitivity—is a long‐standing topic of great interest (Bony & Dufresne, 2005; Bretherton et al., 2013; Brient & Bony, 2013; Brient & Schneider, 2016; Medeiros et al., 2008; Rieck et al., 2012; Vial et al., 2016; Vogel et al., 2016; Webb & Lock, 2013; Wyant et al., 2009; Zhang et al., 2013). Over the recent years, a number of studies have shown that the local interplay between cumulus convection, turbulence, and radiation in the trade wind boundary layer—which manifests at subdaily to longer time scales—is critical for shallow cumulus cloud feedbacks (see Vial et al., 2017, for a review). Beyond their importance for climate sensitivity, modeling studies also show that shallow cumulus clouds have an influence on the large‐scale atmospheric circulation and the overall organization of convection in the tropics. For instance, the ventilation of the boundary layer humidity by shallow convective mixing can affect remotely the structure and strength of the intertropical convergence zone (Gregory, 1997; Neggers et al., 2007; Tiedtke et al., 1988). The longwave emission at shallow cloud tops as well as cold pools from precipitating cumuli impact the temperature and humidity distributions in the lower troposphere, which contribute to the mesoscale organization of shallow convection (Klinger et al., 2017; Naumann et al., 2019), but also to the transition to deep convection (Schlemmer & Hohenegger, 2014) and its aggregation (Coppin & Bony, 2015; Muller & Held, 2012). Therefore, advancing understanding of the key factors controlling (and interacting with) oceanic trade cumuli across a wide range of temporal and spatial scales, not only in models but also in observations, will certainly benefit to our understanding of climate as a whole and our ability to predict it.

One fundamental mode of variability in the tropics is the daily cycle related to the day‐night variations in the solar forcing. These variations can have important effects on climate at longer time scales via nonlinear upscale growth processes (Bernie et al., 2005; Masson et al., 2012; Ruppert, 2016; Ruppert & Johnson, 2016; Shinoda, 2005; Slingo et al., 2003). Several studies have revealed the importance of such feedback mechanisms in the dynamics of the El Niño Southern Oscillation (e.g., Masson et al., 2012) and of the Madden‐Julian oscillation (MJO; Slingo et al., 2003, and references therein). The diurnal modulation in air‐sea fluxes by the solar forcing has long been considered as an important ingredient for upscale growth mechanisms. But more recently, observations from the (Dynamics of the Madden‐Julian Oscillation field experiment have revealed that during the suppressed phases of the MJO, shallow cumulus clouds prevail (with tops below 3 km) over the Indian Ocean of the warm pool region and exhibit a pronounced daily cycle with an afternoon increase in cloud depth (Ruppert & Johnson, 2016). This cumulus convection yields a day‐to‐day heating and moistening that precondition the atmosphere for the transition from a shallow to a deep convective regime (Ruppert, 2016; Ruppert & Johnson, 2016). Hectometer‐scale model experiments have shown that suppressing this cumulus daily cycle can lead to shallower clouds on average over this region and reduced daily‐mean cumulus heating of the troposphere (Ruppert & Johnson, 2016), affecting the time scales for the subsequent transition to deep convection (Ruppert, 2016) and therefore potentially the time scale of the MJO (Slingo et al., 2003).

In general, very distinct daily cycles of cloudiness and associated precipitation have been found depending on cloud types, local environmental conditions, or whether they form over land or ocean. Data collected during the Barbados Oceanographic and Meteorological Experiment and the Atlantic trade wind Experiment have made important contributions to the discovery of a daily cycle of shallow cumulus convection over the trade wind region of the North Atlantic ocean. Observations of cloud covers and kinematic fields have revealed a maximum in cumulus activity at nighttime/early morning and a broad minimum during the afternoon (Brill & Albrecht, 1982; Nitta & Esbensen, 1974). Later, this diurnality has been confirmed with measurements from the Rain in Shallow Cumulus Over the Ocean field experiment, showing a nighttime/early morning peak and an afternoon minimum in precipitation from shallow convection (Nuijens et al., 2009; Snodgrass et al., 2009). Despite these findings, this topic has received very little attention over the past decades, so that our understanding of the diurnal processes in this fair‐weather oceanic cumulus regime and their influence on climate variability at broader scales remains limited. This is also apparent in the reliability of general circulation models, which cannot represent the variability of shallow cumulus and convective processes at subdaily to longer time scales (Nuijens et al., 2015b; Stevens et al., 2019). Ultimately, the representation of the cumulus daily cycle in climate models could potentially also be critical for the estimation of climate sensitivity, as storm‐resolving model (SRM) experiments have shown that the inclusion of a daily cycle of insolation can affect the amplitude of the simulated low‐cloud feedback in the cumulus/transition regimes (Blossey et al., 2009).

**Figure 1:**
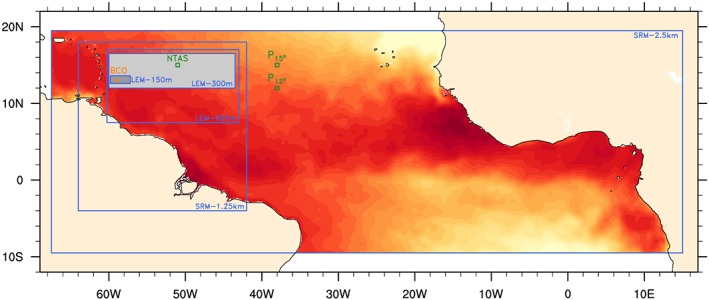
Computational domains of the Icosahedral Nonhydrostatic (ICON) model simulations (blue). The two trade wind domains of analysis are shaded in light and darker gray. Also shown are the locations of surface‐based data sources for Barbados Cloud Observatory (BCO; orange) and for the Prediction and Research Moored Array in the Atlantic (PIRATA) ( $P_{12^{\circ }}$ and $P_{15^{\circ }}$) and Northwest Tropical Atlantic Station (NTAS) buoy sites (green). The color shading in the background is the mean sea surface temperature over the undisturbed Narval period, ranging from about 297 to 302 K. LEM = large‐eddy model; SRM = storm‐resolving model.

In this article, we return to this topic to present a detailed description of the daily cycle of trade wind cumulus convection using modern investigation tools including models and observations. Our analysis focuses on December 2013 when the first *Next‐generation Aircraft Remote‐sensing for VALidation studies*campaign (NARVAL) took place (Klepp et al., 2014; Stevens et al., 2016). Storm‐resolving and large‐eddy simulations have been performed in support of the NARVAL field experiment, using the Icosahedral Nonhydrostatic (ICON) model (Dipankar et al., 2015; Zängl et al., 2015). These simulations are realized under realistic setups on large domains over the tropical Atlantic (Figure 1). This allows for a more consistent representation of the coupling between the boundary‐layer cloud processes and the large‐scale circulation, as compared to most previous large‐eddy model (LEM) studies, which were restrained by the relatively small computational domains (L ≤ 10–50 km) and which mostly used idealized frameworks.

In support of the modeling results, we also present analyses from a range of modern observations for both the short period of the NARVAL field experiment as well as for longer‐term statistics. The rest of the manuscript is structured as follows. Section 2 describes the model simulations and observational data sets used in this study. Section 3 provides a brief description of the simulated clouds and circulation over the North Atlantic trade wind area during the December 2013 Narval period. Sections 4 and 5 describe in detail the daily cycle from the simulations and the observations, respectively. A conclusion and discussion of the results are presented in section 6.

## 2 Model Simulations and Observational Data Sets

We use storm‐resolving and large‐eddy simulations together with a range of in situ and satellite‐based observations. These are described here and documented in Table 1.

**Table 1:** Model Simulations and Observational Data Sets Used in This Study

Model simulations and resolution	Computational domain	Simulated period
SRM‐2.5 km L75	68° W to 15° E, 10° S to 20° N	December 2013
SRM‐1.25 km L75	64° W to 42° E, 4° S to 18° N	December 2013
LEM‐600 m L150	60.25–43° W, 7.5–17° N	11, 12, 14, 15, 16, 20 Dec. 2013
LEM‐300 m L150	60–43.5° W, 8–16.5° N	11, 12, 14, 15, 16, 20 Dec. 2013
LEM‐150 m L150	59.75–57.25° W, 12.6–13.6° N	11, 12, 14, 15, 16, 20 Dec. 2013

Observations	Domain of analysis	Period of analysis
BCO (ground‐based station)	59° W, 13° N	DJF 2012–2017
NTAS (buoy)	51° W, 15° N	December 2013
PIRATA (buoys)	38° W, 15° N and 38° W, 12° N	December 2013
GridSat‐B1 (geostationary satellites)	60–43° W, 12–16.5° N	Dec. 1999–2016 and DJF 2001–2017
CALIOP (polar‐orbiting satellite)	60–43° W, 12–16.5° N	Dec. 2012–2017

### 2.1 ICON‐NARVAL Simulations

We use a range of simulations performed with the ICON model, which include SRM simulations at 2.5‐ and 1.25‐km horizontal grid spacing (hereafter, SRM‐2.5 km and SRM‐1.25 km) and LEM simulations at 626‐, 313‐, and 156‐m horizontal grid spacing (hereafter, LEM‐600 m, LEM‐300 m, and LEM‐150 m).

The SRM was run with a stretched vertical grid of 75 levels up to 30 km, including 25 levels within the first 2,500 m and with a grid spacing of about 130 m at 920 m height. The model has the same physical package as the code used for the operational numerical weather prediction, except that the parameterizations for shallow and deep convection, gravity wave drag, and subgrid‐scale orography were switched off. Diagnostic cloud fraction is calculated using a simple box probability density function of total water content, assuming a fixed variance around the gridbox mean value (Daniel Klocke, personal communication, May 2, 2019). More information on the model characteristics is given in Zängl et al. (2015) and Klocke et al. (2017).

The LEM differed from the SRM in that it was run with 150 vertical levels (with a grid spacing of about 80 m at 930 m height) and used different physical parameterizations for turbulence and microphysics, as described in Dipankar et al. (2015) and implemented in Heinze et al. (2017). In addition, no subgrid‐scale cloud scheme was used. Cloud fraction is 1 if the liquid water content is greater than 10^−5^ kg/kg, and 0 otherwise.

The SRM with 2.5‐km grid spacing was run every day of December 2013 over the entire tropical Atlantic (Figure 1). The simulations were initialized each day at 0000 UTC with analysis data—including assimilated NARVAL dropsondes—from the European Centre for Medium‐Range Weather Forecasts and integrated for 36 hr. The results are analyzed and presented at 1‐hr frequency from 1300 to 1200 UTC (the following day), after 12 hr of spin‐up. Lateral boundary data from European Centre for Medium‐Range Weather Forecasts analysis were nudged at 3‐hourly intervals, except for the sea surface temperature (SST) fields, which were kept constant at their initial values throughout the 36‐hr simulations. Although the surface is usually an important factor for the daily cycle over land and even sometimes over the ocean (Ruppert & Johnson, 2016), the SST in the trade winds undergoes very little diurnal variations (as will be discussed in the next section) and as such appears to be a negligible factor in the daily cycle studied here.

These 2.5‐km runs also include the 1.25‐km nested SRM simulations over the western Atlantic (Figure 1), which in turn were used as input to the LEM simulations on smaller domains around Barbados (Figure 1). The LEM simulations were performed for selected days during December 2013 (11, 12, 14, 15, 16, and 20), to match the flight operations of the NARVAL campaign. Output from SRM‐1.25 km were used for the initialization (at 0900 UTC each day) and the nudging of the lateral boundaries (every hour). The model was integrated for 27 hr, and the output is analyzed and presented at 15‐min frequency from 1300 to 1100 UTC (the following day), after 4 hr of spin‐up (23‐hr days are used to match the beginning of the days in the SRM).

**Figure 2:**
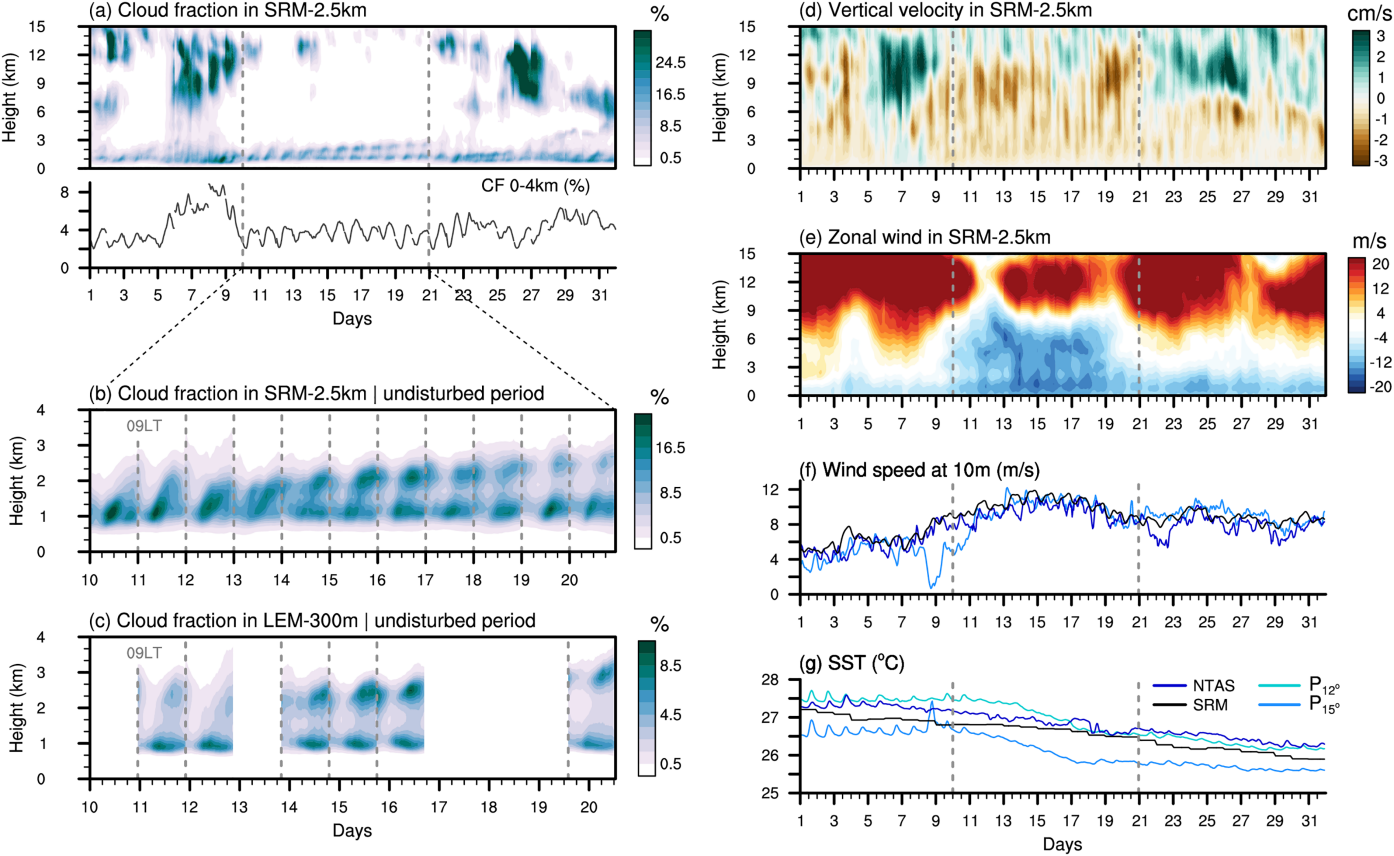
Hourly time series of cloudiness and circulation over the large trade wind domain during December 2013. (a) Time‐height series of cloud fraction as simulated by SRM‐2.5 km over the whole month (top) along with the averaged cloud fraction between 0 and 4 km (bottom). (b) Same as the upper panel of (a) but zooming in the lower troposphere and on the undisturbed period, from 10 to 20 December 2013. (c) Same as (b) but for the 6 days available using LEM‐300 m. Time‐height series of (d) vertical velocity and (e) zonal component of the horizontal wind using SRM‐2.5 km. Time series of (f) near‐surface wind speed at 10 m and (g) SST, as simulated by SRM‐2.5 km (black line) and observed at the three selected buoy sites (blue lines) in the North Atlantic trade wind area shown in Figure 1 (NTAS; P ${}_{12^{\circ }}$, P ${}_{15^{\circ }}$). LEM = large‐eddy model; SRM = storm‐resolving model; SST = sea surface temperature.

Note that the daily initialization creates a day‐to‐day discontinuity that is not completely smoothed even after the spin‐up period (Figures 2a–2c and S1 in the supporting information). This discontinuity is more or less strong depending on days and variables, but overall, it slightly artificially amplifies the diagnosed diurnal variations. If one was designing a set of simulations to identify a diurnally‐varying signal in cloudiness, the simulations would run over a longer period than used here to allow the diurnal signal to emerge from longer‐term variability. While that is not the case in the present setup, the simulated daily cycle does not occur every day, and its sporadic occurrence is consistent with the large‐scale environmental conditions in which the clouds form (section 3). This supports the fact that there is a physical basis for this diurnal signal and that it is not merely the result of spin‐up artifacts. Moreover, and as will be shown in section 5, the observations, which do not suffer from spin‐up effects, exhibit a similar daily cycle as the model, so that overall, further study of the details of this daily cycle in these simulations is relevant and worthwhile.

Our analysis of the daily cycle is carried out on two domains. The bigger one covers about 1,900 × 500 km^2^ in the trade wind area, which includes Barbados (light gray shading in Figure 1). This domain is part of the computational domain for all simulations except for LEM‐150 m, which was run on a smaller domain. Unless otherwise stated, this bigger domain is our main domain of analysis. A second, smaller domain is also considered when the comparison is made with LEM‐150 m; it covers an area of about 300 × 100 km^2^ that also includes Barbados (dark gray shading in Figure 1).

### 2.2 Observations

Ground‐based radar, ceilometer, and Micro Rain Radar (MRR) measurements from the Barbados Cloud Observatory (BCO)—located on a windward promontory on Barbados (59.48° W, 13.15° N, see Figure 1)—are used to derive cloud and precipitation statistics for the winter season from 2012 to 2017 (December–February [DJF]). All data are here binned into 5‐min averages. A detailed description of the BCO instrumentation can be found in Nuijens et al. (2014) and Stevens et al. (2016).

The MRR is used to compute both the rain rate, which is taken at the lowermost level with trustworthy data (i.e., 320 m), and the rain mask, which is set to 1 when rain rates exceeding 0.05 mm/hr are measured in at least five 30‐m range gates within the lowest 3 km. The rain mask is used to derive the rain frequency.

The fraction of hydrometeors (cloud and rain droplets) is obtained from two 36‐GHz Doppler cloud radars, using all radar returns with an equivalent radar reflectivity larger than −40 dBZ. Additionally, profiles of cloud fraction are obtained by correcting for periods of rain. Thereby, periods are disregarded if (a) the ceilometer does not detect a cloud base due to strong rain or (b) the MRR rain mask is 1. Furthermore, radar returns below the ceilometer‐detected cloud‐base height are set to 0. Periods where neither the ceilometer nor the MRR are available are discarded in the rain‐corrected radar cloud fraction.

The ceilometer is also used to derive a measure of total cloud cover, defined as the fraction of time that a cloud‐base is detected. Periods when the ceilometer does not detect a cloud base while the MRR sees rain are discarded from the analysis. Moreover, we separate the contribution of cloud bases that are detected below or above 1 km (as in Nuijens et al., 2014).

To make sure that synoptic disturbances or sporadic deep convection do not obscure the diurnal cycle, we further discard periods with a radar signal above 4 km (including the previous and following hour). We thus limit ourselves to periods of shallow convection with only little rain.

We also use SST and near‐surface winds for December 2013 as measured at selected buoys in the tropical Atlantic (Figure 1). These include the Northwest Tropical Atlantic Station (NTAS) (at 51° W, 15° N) and two stations from the Prediction and Research Moored Array (PIRATA) in the Atlantic network (at 38° W, 15° N and 38° W, 12° N).

Finally, satellite‐based retrievals are used to provide statistics on the larger scale—on domains that are comparable to those used in the models.

The Gridded Geostationary Satellite product from the International Satellite Cloud Climatology Project B1 data set (GridSat‐B1) provides calibrated and gridded data from imagers on satellites worldwide, which are sampled to approximately 8‐km resolution and 3‐hourly intervals (Knapp et al., 2011). We use infrared brightness temperature (T_*b*_) in the 11‐μm channel to detect shallow cloud tops whenever 280 K ≤ T_*b*_ ≤ 290 K. The total area covered by shallow clouds over the large trade wind domain (cf. Figure 1 and Table 1) is estimated for the winter season from 2001 to 2017.

The Cloud‐Aerosol Lidar and Infrared Pathfinder (CALIPSO; Winker et al., 2009) and CloudSat (Stephens et al., 2002) satellites, flying in the A‐train constellation since April 2006, provide 3‐dimensional cloud profiling from radar and lidar. CALIOP is very sensitive to clouds and provides very accurate cloud heights but is unable to see below clouds with optical depths larger than about 3 (i.e., thick water clouds mostly). CloudSat carries a W‐band cloud‐profiling radar but is much less sensitive than the cloud radars at BCO. In principle, CloudSat profiles would be useful in identifying clouds near the lifting condensation level lying underneath higher clouds opaque to the CALIOP lidar, but CloudSat fails to detect many low‐altitude shallow clouds (Liu et al., 2016). Therefore, this study uses only CALIOP observations. Shallow clouds with tops below 4 km height are sampled over the large trade wind domain (cf. Figure 1) for the December months from 2012 to 2017. The A‐train orbit is Sun‐synchronous, crossing the equator at approximately 01:30 and 13:30 local times, with the same ground track repeated in a 16‐day cycle. The daily cycle is therefore only sampled twice per day. As will be shown in section 5.2, however, these two times almost coincide with the times of minimum and maximum in the daily cycle of cloud amount.

## 3 General Characteristics of Trade Wind Cloudiness and Circulation

Figure 2 illustrates the temporal evolution of cloudiness and circulation over the North Atlantic trade wind domain (light‐gray area in Figure 1) as simulated by SRM‐2.5 km and LEM‐300 m during December 2013. Two distinct weather regimes are depicted: two convectively active periods (4–9 and 22–31 December 2013), characterized by the presence of clouds at all levels and large‐scale tropospheric ascent, and one undisturbed period (10–20 December 2013) with subsiding motion throughout the troposphere and shallow convective clouds with tops at 3–4 km.

Diurnal variability in low‐level clouds with a pronounced day‐to‐day recurrence is clearly apparent during the undisturbed period. The results show a nighttime cloud deepening and overall increase in cloudiness followed by a daytime reduction (Figures 2a–2c). This daily cycle is comparable between the model versions (despite some resolution‐dependent features, which are discussed in the next section) and is consistent with previous observational findings focused on a similar trade wind region (Brill & Albrecht, 1982; Nitta & Esbensen, 1974; Nuijens et al., 2009).

Figure 2e shows that the daily cycle occurs exclusively within strong easterly winds near the surface and in most of the troposphere (up to 9–10 km). During the rest of the month, and especially at the time of deep convective cloud systems (e.g., 6–8 December), the upper‐tropospheric westerlies extend far downward in the free troposphere, presumably due to downward westerly momentum transport by convection. Wind speed has a strong influence on ocean surface temperature variations. In the trade winds, the SST is generally expected to undergo very slight diurnal variations (Brill & Albrecht, 1982), because of strong wind mixing—and with it weak solar warming—of the oceanic surface layer (Bellenger et al., 2010; Halpern & Reed, 1976; Stramma et al., 1986). This relationship between diurnal SST variations and near‐surface wind speed is nicely depicted in the observed time series from buoys (Figures 2f and 2g): First, in the convectively active period from 1 to 8 December, the wind speed is relatively weak (<8 m/s), and the SST undergoes a weak daily cycle (of about 0.5 K); then when the wind strengthens during the undisturbed period (up to 9–12 m/s), the SST drops, and the diurnal variations vanish. The SST is thus likely to play a minor role in the daily cycle of oceanic shallow convection in the trade winds. This is not the case, however, with the cumulus daily cycle over the warm pool region found in Ruppert and Johnson (2016), which rather occurs during periods of weak near‐surface winds (in the suppressed phases of the MJO). In that case, the ocean surface layer undergoes a pronounced solar warming, leading to daytime increases in surface fluxes, which in turn have a similar effect as over land in promoting the development of clouds during the afternoon. Therefore, depending on the environmental conditions, distinct daily cycles of shallow cumulus convection over the ocean can occur. In the following section, we document the diurnal characteristics of oceanic shallow convection in the trade wind region near Barbados, with a focus on cloudiness characteristics and precipitation during the undisturbed Narval period.

## 4 Simulated Daily Cycle

### 4.1 Results From a Realistic Large‐Domain LEM

**Figure 3:**
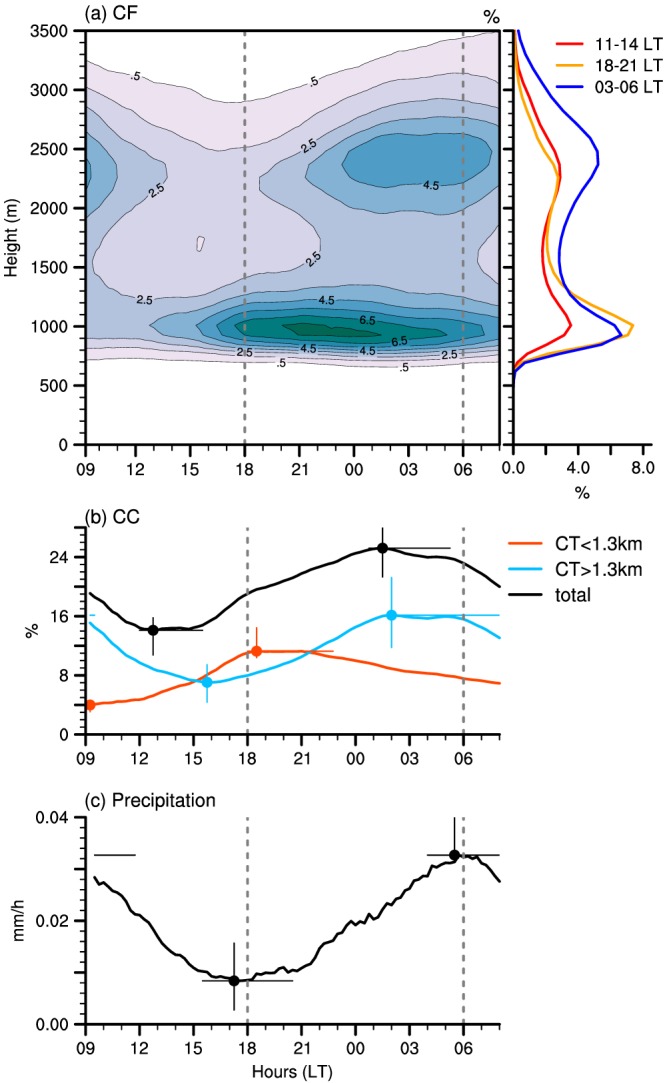
Mean diurnal characteristics of the trade wind cloudiness and precipitation over all simulated days from LEM‐300 m: (a) vertical distribution of cloud fraction and averaged profiles for selected times; (b) total cloud cover (black) and contributions from clouds with cloud top below 1.3 km (orange) and above 1.3 km (cyan); (c) surface precipitation. The large dots in panels (b) and (c) show the averaged values for the daily minimum and daily maximum. The error bars correspond to the range of daily extrema (vertical bars) and local times of daily extrema (horizontal bars) over the six simulated days. LEM = large‐eddy model; CF = cloud fraction; CC = cloud cover.

The diurnal characteristics averaged over all simulated days for LEM‐300 m (cf. Figure 2c) are shown in Figure 3. They clearly reveal a weakening of the shallow convection during the day and a strengthening overnight. A decrease in overall cloudiness in the morning, reaching a minimum in the early afternoon (between 12 and 15 LT), is followed by a significant increase in cloud cover and cloud depth until early morning hours before sunrise. The total cloud cover has a diurnal amplitude of about 10% with a minimum of ∼15% between 12 and 15 LT and a maximum of ∼25% between 01 and 05 LT (black curve in Figure 3b). The two maxima in cloud fraction represented by the model near cloud base and under the trade inversion (Figure 3a) constitute a common feature of shallow cumulus clouds in the region of Barbados (Nuijens et al., 2014, 2015b). They both undergo a pronounced diurnal variation, but with a significant phase lag. The low‐level cloud fraction around 1‐km peaks between 21 and 00 LT, while clouds aloft, centered around 2.5 km, have their peak frequency just before sunrise (profiles in Figure 3a). These two maxima in cloud fraction can reflect two separate layers of clouds (one centered around 1 km and one around 2.5 km), or they can be associated with clouds that cover the whole vertical extent.

To gain more insights into these two frequency maxima, we define different cloud types and analyze their relative contribution to total cloud cover (Figure 3b and Appendix A). In essence, we follow the idea of Nuijens et al. (2014), although with slightly different definitions as detailed below and in Appendix A.

**Figure A1:**
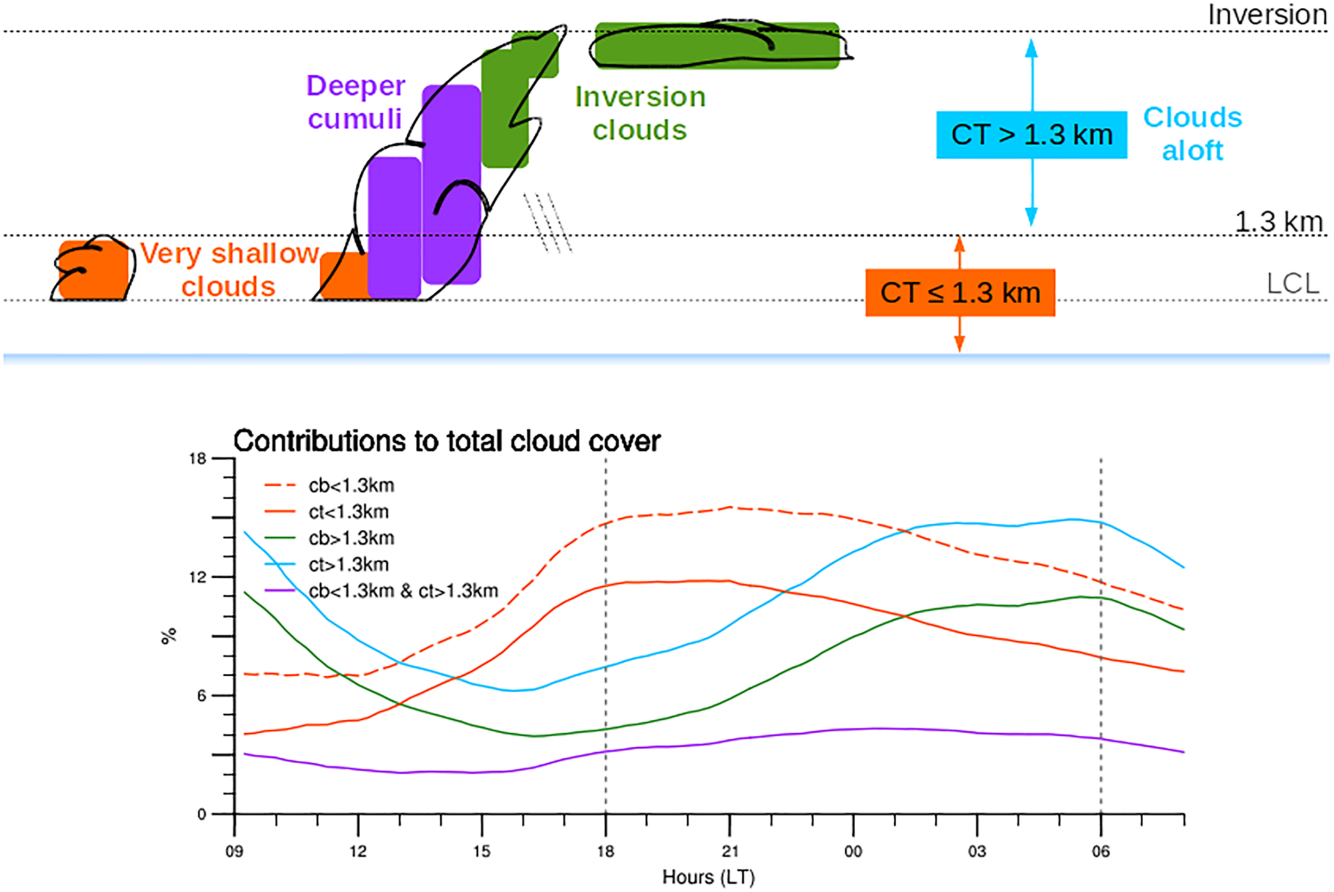
Schematic of the different cloud contributions to total cloud cover (top panel) and their corresponding daily cycle (bottom panel, solid lines) for LEM‐300m. The dash line in the bottom panel is for clouds with cloud‐base below 1.3 km.

We consider a first category constituted of *very shallow clouds* with little vertical development (orange curve in Figure 3b). These clouds typically form at the top of the mixed layer and are defined here to have their top below 1.3 km (so as to include the lowermost peak frequency seen in Figure 3a). These very shallow clouds might be referred to as the Cumulus Humilis (C_*L*_ = 1) from the cloud atlas of the World Meteorological Organization (https://cloudatlas.wmo.int), although strictly speaking, here they may also be the shallow part of a tilted cloud, as illustrated in Figure A1.

Once the very shallow clouds have formed, they can grow deeper, be leaned by horizontal wind shear, spread laterally under the trade inversion—to form what may be called a stratiform cloud shield or shallow anvil—or separate from the cloud core. We thus consider another category to account for these more developed cumulus, defined here to have tops above 1.3 km (cyan curve in Figure 3b). Note that we do not set a criterion for the height of the cloud base for this second category. However, Figure A1 shows that clouds with cloud base above 1.3 km dominate the diurnal variation of this cloud category. As will be shown later, this category best reflects the stratiform layer of clouds below the trade inversion; following the World Meteorological Organization cloud atlas, we would call these clouds the Stratocumulus Cumulogenitus (C_*L*_ = 4). Unless otherwise stated, we hereafter simply refer to this category as the *clouds aloft*. The total cloud cover (black curve in Figure 3b) corresponds to the sum of the two categories: the contribution of very shallow clouds (orange) and that of clouds aloft (cyan).

**Figure 4:**
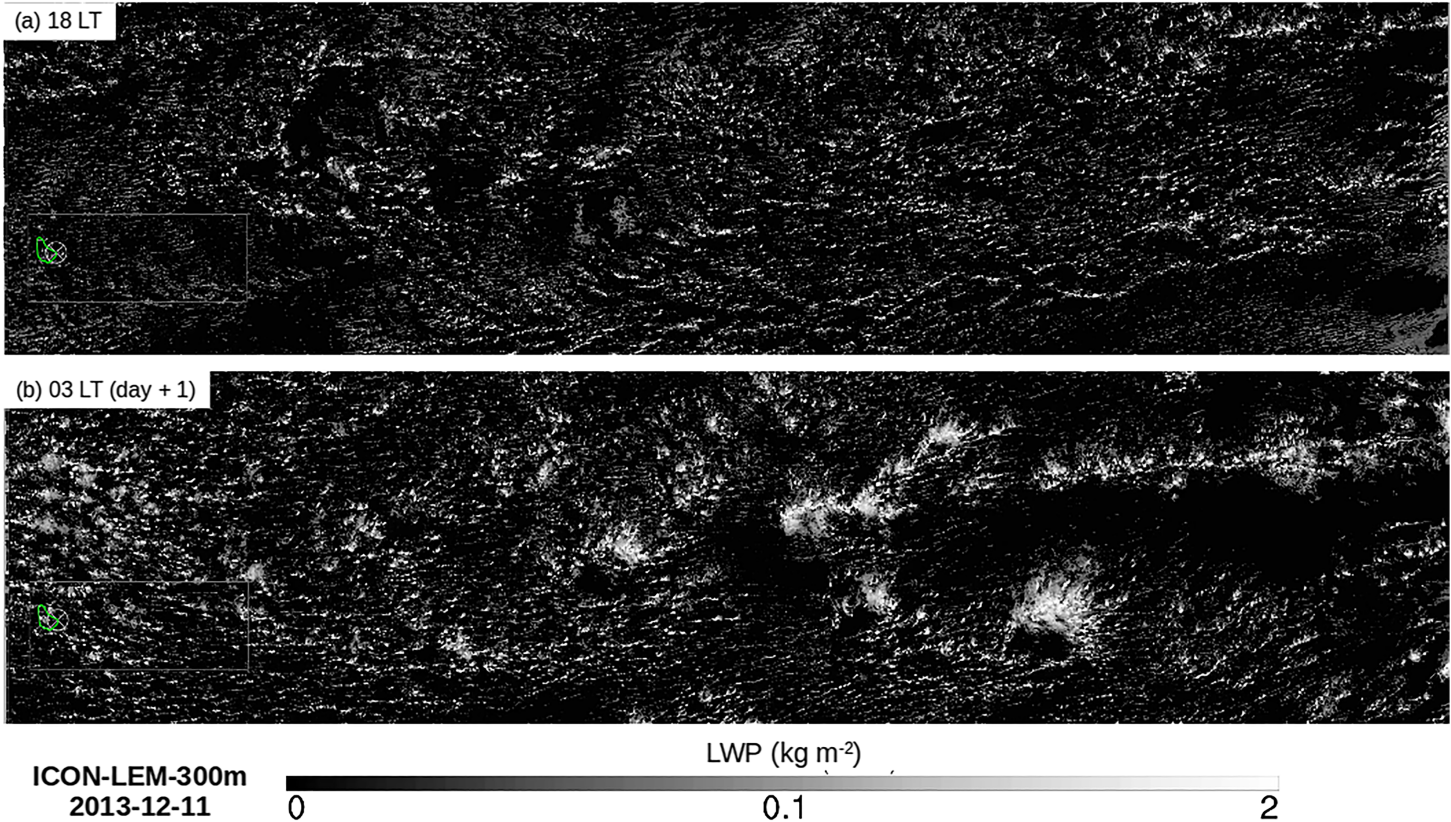
Snapshots of liquid water path from LEM‐300 m on the 11 December 2013 at (a) 18 LT and (b) 03 LT (day + 1). Figures produced by Matthias Brueck. LEM = large‐eddy model; ICON = Icosahedral Nonhydrostatic; LWP = liquid water path.

The peak in cloud fraction around 1 km (Figure 3a) is largely tied to the variability of the very shallow clouds and, at a lesser extent, to that of the deeper trade cumuli (Appendix A). The contribution of the very shallow clouds to cloud cover increases during daytime, peaks after sunset (between 18 and 21 LT), and slowly decreases throughout the overnight period (orange curve in Figure 3b). These clouds have an area coverage about three times higher around sunset (∼12%) than in the morning at 09 LT (∼4%), and they are the dominant type of clouds over the domain from about 15 to 22 LT. This is also apparent in the horizontal distribution of the liquid water path at 18 LT on the 11 December 2013 (Figure 4a), which reveals a multitude of small and thin clouds regularly scattered over the whole domain. At the time when this cloud population dominates, surface precipitation is minimum (Figure 3c), and the associated pattern of mesoscale organization is reminiscent of the “Sugar” pattern introduced in Stevens et al. (2019; so named as it is evocative of the dusting of powder sugar).

At the end of the afternoon, the clouds start to grow deeper, as evidenced by the increase in the contribution of clouds aloft at around 16 LT (blue curve). This cloud population dominates most of the time and undergoes a pronounced diurnal variation as well. The area coverage associated with clouds aloft depicts a minimum of ∼7% at 16 LT, followed by a maximum of ∼16% between 02 and 05 LT (a twofold variation). The nighttime cloud field in Figure 4b appears with a brighter contrast as the clouds have now reached a greater vertical extent. Some amount of stratiform‐like clouds, contributing to the total cloud cover, is also visible. The precipitation diurnal variation is clearly connected to the evolution of this cloud category (compare Figure 3c with the blue curve in Figure 3b). It is minimum before sunset and maximum around sunrise, which is consistent with observations during the Rain in Shallow Cumulus Over the Ocean campaign (Nuijens et al., 2009). As Figure 4b suggests, the increased precipitation overnight leads to an enhanced cold pool activity and associated gust fronts along which secondary convection can form.

### 4.2 Sensitivity to Model Resolution and Analysis Domain

**Figure 5:**
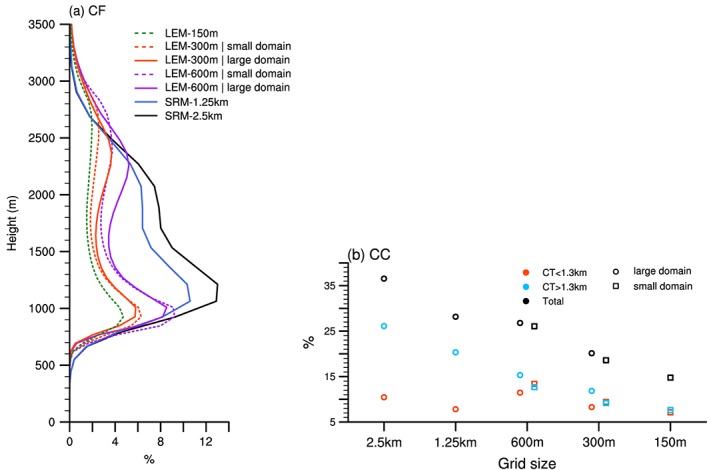
Daily‐mean cloud fraction for all simulated days within the undisturbed Narval period (10–20 December 2013) from the different model versions of Icosahedral Nonhydrostatic. The solid line corresponds to spatial average over the large domain, and the dash line is for the small domain (cf. Figure 1). LEM = large‐eddy model; SRM = storm‐resolving model.

The daily cycle presented above is consistent among all model versions, although there are a few notable differences. Some of them are not specific to the daily cycle but rather relate to the overall representation of clouds in the model. It is thus useful to first consider daily‐mean cloud properties as presented in Figure 5.

In the LEM, in general, clouds have a greater vertical extent than in the SRM (Figure 5a). The first peak frequency near cloud base is ∼200 m lower, while the peak under the trade inversion is ∼200 m higher, in the LEM than in the SRM. Comparison is also made between the small and large domains that are considered for the analysis (cf. Figure 1). The averaged cloud population is deeper over the small domain (dash lines) than over the larger domain (solid lines), which suggests that clouds with more variable depths can be captured when considering the larger domain. This might reflect a westward deepening of the clouds as the SSTs increase westward (Figure 1).

**Figure 6:**
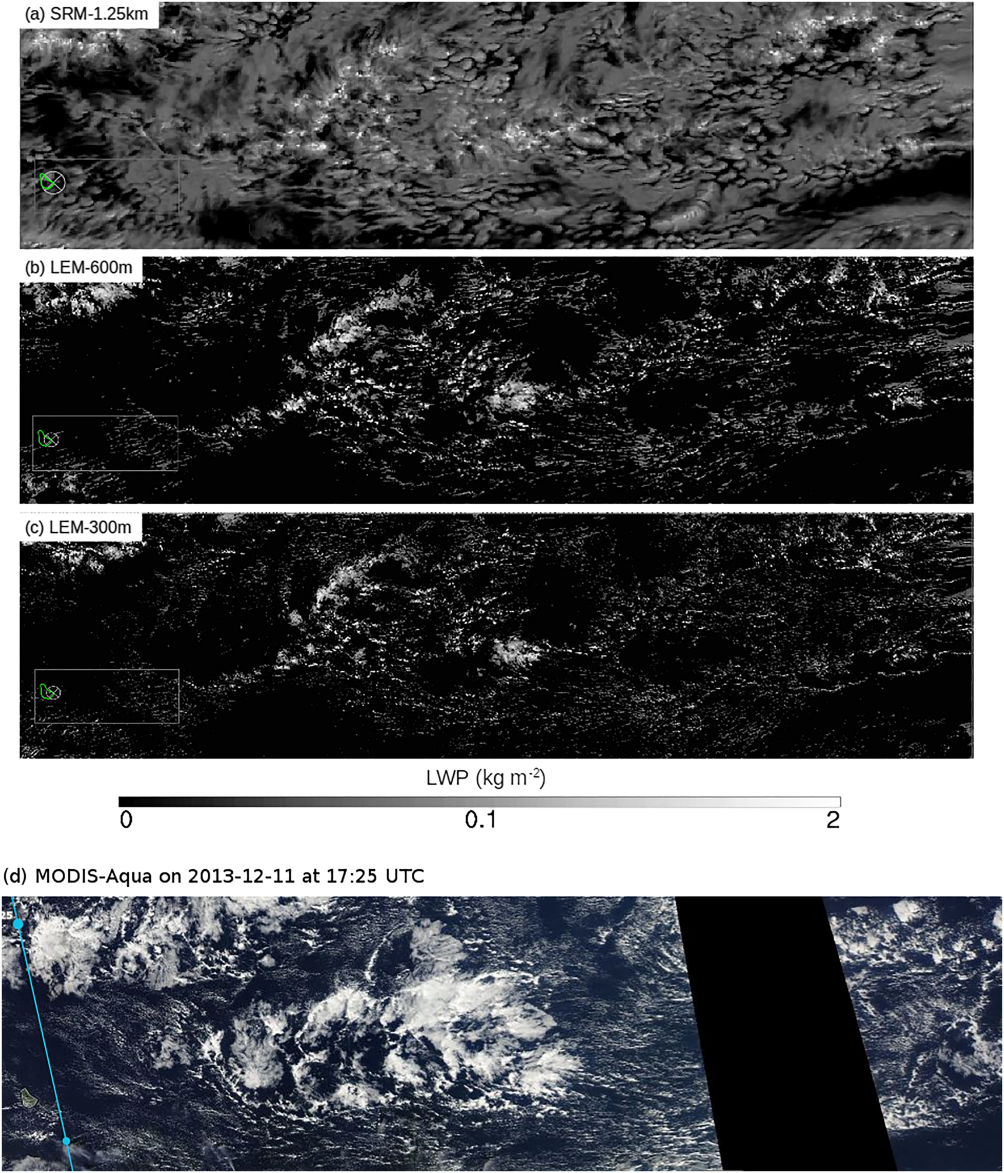
Snapshots of liquid water path for (a) SRM‐1.25 km, (b) LEM‐600 m, and (c) LEM‐300 m on 11 December 2013 at 13:25 LT (17:25 UTC). Figures produced by Matthias Brueck. Panel (d) shows the corresponding MODIS‐Aqua scene from Worldview, with orbital track upstream Barbados shown in blue (the small dot in the lower left corner indicates 17:24 UTC). LEM = large‐eddy model; SRM = storm‐resolving model.

A significant decrease in cloud fractions and total cloud cover is also notable as the model resolution is increased, both in SRM and LEM. When comparing the two SRM simulations on one hand, and the SRM against LEM simulations on the other hand, the decrease in total cloud cover with respect to grid resolution largely comes from a reduction of clouds aloft (Figure 5b), and more specifically clouds with cloud base above 1.3 km (e.g., the stratiform outflow—not shown). In the LEM, however, both the contributions of the very shallow clouds and the clouds aloft contribute more or less equally to the differences in total cloud cover (compare for instance the squared markers in Figure 5b). This result is also visible from the horizontal distributions of liquid water path in Figure 6, especially when comparing SRM‐1.25 km and LEM‐600 m. In SRM‐1.25 km, most of the domain is covered by a thin layer of clouds, as evidenced by the widespread gray color in Figure 6a. Such a feature is, however, not reproduced in the LEM (Figures 6b and 6c). This, together with the results of Figure 5b, therefore shows that at lower resolution, the model produces much more stratiform clouds below the trade inversion.

Other studies have investigated the sensitivity of trade wind cumulus clouds to model resolution in LEMs and have reported an increased cloud cover with smaller grid spacing (Matheou et al., 2011; Sato et al., 2018), while in ICON, the resolution‐dependency is opposite. The reasons are unknown, although the studies mentioned above have employed very different experimental protocols (smaller grid sizes, smaller computational domains, and idealized boundary conditions). Using a similar range of resolutions as here, Blossey et al. (2009) have found a reduction in cloud fractions and shortwave cloud forcing with smaller grid spacing, which is in better accordance with the results of this present study. It could be argued that the resolutions employed here are insufficient to represent shallow convection. The simulations are yet performed on very large domains with realistic boundary conditions. These characteristics are important to represent the coupling between clouds and the large‐scale circulation and to allow larger‐scale mesoscale organization of shallow convection as observed in nature (Stevens et al., 2019). Whether or not these ICON simulations can realistically represent the cloud features can be appreciated by comparing the model snapshots with the corresponding MODIS‐Aqua picture (Figure 6). It suggests some consistency in terms of the dependence of cloudiness on large‐scale factors (e.g., humidity and winds). For instance, all simulations can fairly well reproduce the dry areas depleted of clouds in the lower left and lower right parts of the domain. The presence of a more developed cloud structure in the center of the domain is also captured, although the details of the observed pattern of organization is hardly reproduced in any of the simulations. One striking feature is that the SRM overestimates the stratiform cloud type as compared to the MODIS picture, while the LEM tends to underestimate it. In the LEM, the pattern of very small and thin clouds, located upstream of the large cloud structure, can be captured, however, and more so as the resolution is increased. Therefore, all simulations can reproduce fairly realistic cloud patterns at large scale, but at mesoscale and cloud‐scale the model still suffers from large discrepancies with respect to the observations, especially as the resolution is decreased.

**Figure 7:**
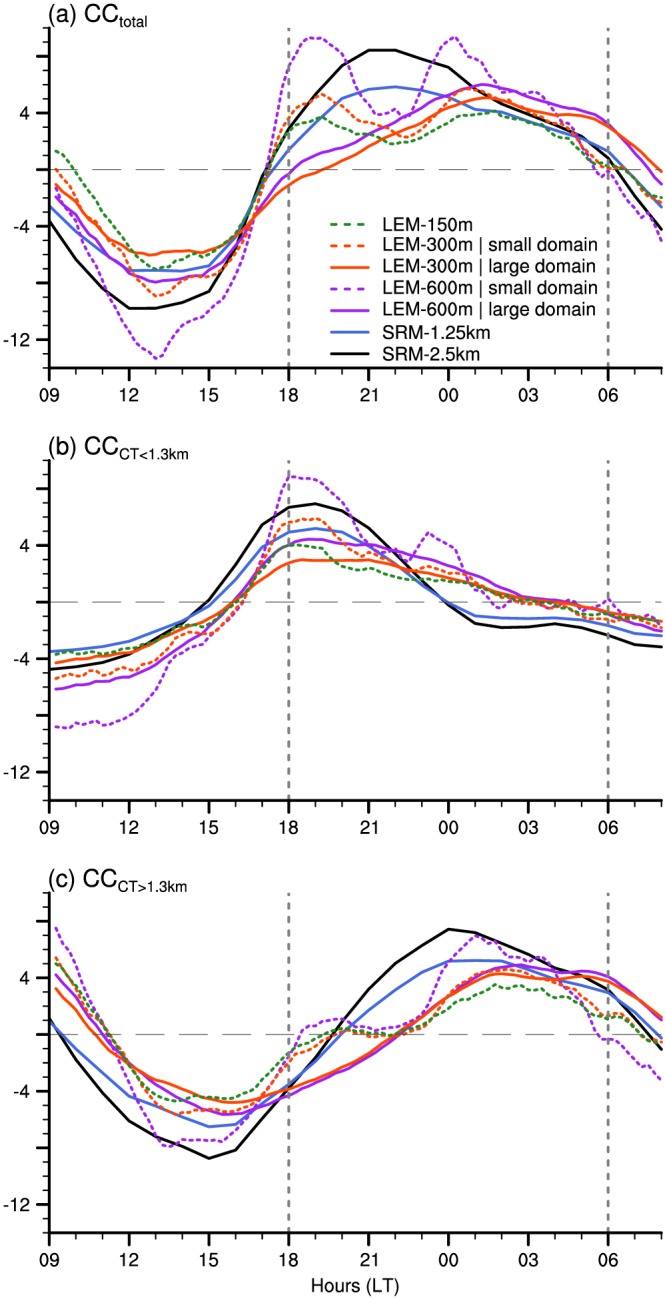
Mean daily cycle over the undisturbed period from all model simulations for (a) the total cloud cover and its contribution from (b) low‐level clouds with tops below 1.3 km and (c) clouds with tops above 1.3 km—as in Figure 3b but with the daily mean subtracted. As in Figure 5, the solid line corresponds to the large domain averages and the dash line to the small domain. Note that all cloud covers are halved for the SRM simulations, so as to fit on the same scale. LEM = large‐eddy model; SRM = storm‐resolving model.

Model uncertainties that are more specific to the daily cycle are now discussed with the aid of Figure 7. Both the model resolution and the choice of the analysis domain affect the diurnal characteristics of trade wind clouds. When using the smaller domain (dashed lines), the diurnal evolution of total cloud cover exhibits a more pronounced minimum in the early afternoon, followed by two maxima of similar amplitude, as compared to when using the larger domain (solid lines). The first peak occurs just after sunset and is related to a stronger increase in cloud cover from both the very shallow clouds and the clouds aloft (Figures 7b and 7c). The second peak (after midnight) and the stronger midday reduction in cloudiness are, however, essentially connected to a more pronounced variation of clouds aloft.

The phase and the amplitude of the daily cycle are also affected by the type of models used and the resolution. Overall, the diurnal amplitude depends on the amount of clouds and thus on the model resolution: the greater the cloud cover (when resolution is reduced), the more pronounced the daily cycle. The other notable difference is in the exact timing of the nocturnal peak in cloud cover, which occurs about four hours later in the LEM (around 01 LT) than in the SRM (around 21 LT; Figure 7a). This difference is traced back to clouds alofts (Figure 7c), which seem to develop more slowly in the LEM than in the SRM—i.e., the overnight increase is weaker in ICON‐LEM than ICON‐SRM.

In general, most of the uncertainties in high‐resolution simulations of trade wind cumulus clouds arise from the amount of stratiform clouds below the trade inversion, while cloud fractions near cloud‐base appear more consistent among the various studies (Vial et al., 2017, and references therein). Interestingly, the results presented in this section lead to the same conclusion: Clouds aloft are largely responsible for uncertainties in the averaged total cloud cover and in the time of its nocturnal peak. In the next section, we present the results from observations, which will help clarify which features are more realistic.

## 5 Observed Daily Cycle

### 5.1 BCO Measurements

**Figure 8:**
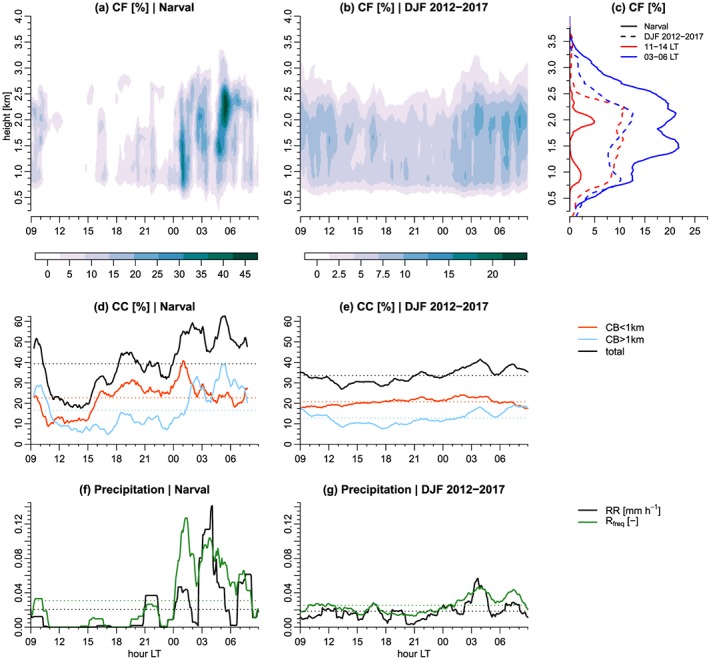
Observed mean daily cycle of cloudiness and precipitation from the Barbados Cloud Observatory. (Top row) Rain‐corrected cloud fraction derived from the radar for (a) the undisturbed Narval period and (b) the climatology (December–February [DJF] 2012–2017), and (c) profiles at selected times for Narval (solid) and climatology (dashed), (middle row) ceilometer‐derived cloud cover for (d) Narval and (e) climatology, and (bottom row) Micro Rain Radar derived rain rate (RR) and rain frequency (R_freq_) for (f) Narval and (g) climatology. The data in all panels are smoothed with a 1.5‐hr centered linear filter. The thin dotted lines in panels (d–g) represent the daily mean. Note that the color scale is different for the climatology and for the Narval period (a and b) and that the rain frequency and rain rate share the same *y* axis (f and g).

Measurements at BCO have made important contributions to our knowledge of the trade wind cumulus cloud variability in the region of Barbados (Brueck et al., 2015; Lamer et al., 2015; Nuijens et al., 2014, 2015b). However, the daily cycle has never been documented yet. It is here presented in Figure 8 and Table 2 for the undisturbed Narval period (10–20 December 2013), as well as for the DJF climatology between 2012 and 2017. Over the 10‐day period of December 2013, all instruments depict a daily cycle with very similar characteristics as those discussed for the ICON simulations (section 4.1), at least in a qualitative sense. Radar‐derived cloud fractions are about four times higher at nighttime (between midnight and 06 LT) than during daytime (11–16 LT in Figures 8a and 8c, solid lines). Important variations occur from cloud‐base (below 1 km) to cloud top, and the overnight deepening of the cloud layer is also clearly depicted. Comparing the radar echo fraction profiles with and without rainfall correction (see section 2.2) provides an estimation of rainfall frequency in the sub‐cloud layer that is about 10% during nighttime and near zero during the day (Figure S2c). This is also consistent with the MRR measurements, which show a greater rainfall rate and frequency between midnight and early morning hours before sunrise than during daytime (Figure 8f). The ceilometer‐detected total cloud cover has a diurnal amplitude of about 30%, with a minimum of ∼25% at 15 LT and a maximum of ∼55% at 03 LT (Figure 8d and Table 2). Both cloudiness detected below 1 km and cloudiness aloft contribute significantly to this diurnal variation in cloud cover (Figure 8d and Table 2). A first peak in cloudiness near cloud base occurs quite soon after sunset, followed by a second peak—presumably associated with deeper cumuli (see Appendix A)—around midnight, while the nocturnal development of the stratiform cloud layer below the trade inversion reaches its maximum at early morning hours before sunrise (Figure 8d, compare orange and cyan lines).

**Table 2:** Observed Diurnal Characteristics of Cloudiness From BCO—Amplitude (in %) and Local Time (Rounded to the Nearest Hour) of the Daily Minimum and Maximum in Cloud Cover—as Derived From the First Harmonic of the Mean Daily Cycle for (Left) the Narval Period and (Right) the Climatology (DJF 2012–2017)

	Minimum		Maximum	
	NARVAL	DJF 2012‐2017	NARVAL	DJF 2012‐2017
CC	25.4 (15 LT)	29.3 (16 LT)	53.4 (03 LT)	37.8 (04 LT)
CC_*CB* ≤ 1km_	15.0 (12 LT)	18.6 (12 LT)	30.5 (00 LT)	22.9 (00 LT)
CC_*CB*>1km_	6.2 (17 LT)	9.0 (18 LT)	27.1 (05 LT)	16.6 (06 LT)

*Note*. BCO = Barbados Cloud Observatory; DJF = December–February; NARVAL = Next‐generation Aircraft Remote‐sensing for VALidation studies campaign.

The comparison between the observations and the simulations, therefore, turns out favorably for the LEM, as it best represents the early morning peak in cloudiness aloft (cf. Figure 7). Note that despite different definitions used to classify the trade wind cumulus in the models and in the observations (i.e., according to cloud base or cloud top height), the two cloud populations discussed here—the very shallow clouds and clouds aloft—are comparable between the models and the observations (see Appendix A).

### 5.2 Satellite‐Based Retrievals

**Figure 9:**
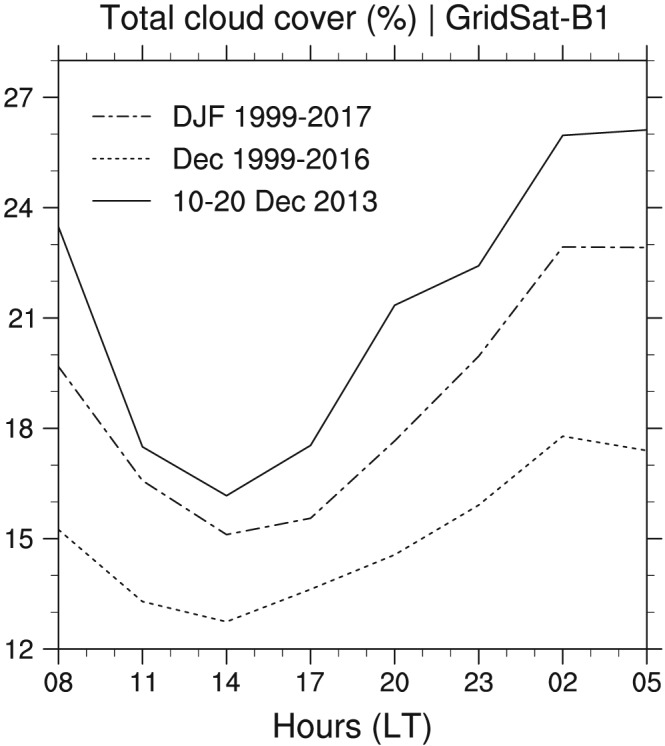
Observed mean daily cycle of total low‐cloud cover from GridSat‐B1 retrievals for three different periods: 10–20 December 2013 (solid), December 1999–2016 (dash dotted) and December–February (DJF) 2000–2017 (dotted).

Using the GridSat data (section 2.2) over this same period but a larger domain area, we found a similar daily cycle in total low‐cloud cover, with a minimum of ∼15% at midday and a maximum of ∼25% just before sunrise (Figure 9). However, we note that the values of the total low‐cloud cover and the diurnal amplitude are much lower in GridSat than in BCO, especially at nighttime when the cloud amount is larger (the difference between the two data sets ranges between ∼10% at 15 LT and ∼25% at 03 LT). The total low‐cloud cover as measured by CALIOP is 26% at 13:30 and 46% at 01:30 local times (Table 3), which is in much better agreement with the BCO values at these times (cf. Figure 8d). However, the detection of shallow cloud tops with CALIOP underestimates the cloud cover from the very shallow clouds and overestimates that from clouds aloft, as compared to the cloud‐base detection at BCO (by a factor 2 during the day and by a factor 3 or 4 at nighttime). This difference can be understood by the different cloud classification methods used with BCO and CALIOP (i.e., cloud base vs. cloud top separation; see Appendix A). Moreover, with CALIOP the signal is easily attenuated by water clouds aloft, preventing the detection of lower cloud layers, while the ceilometer tends to do the opposite—its signal is more easily attenuated by clouds near cloud base, which prevents the detection of clouds aloft.

**Table 3:** Day/Night Cloud Covers (in %) Estimated From CALIOP Over the Large Trade Wind Domain

	Day: 13:30 LT		Night: 01:30 LT	
	NARVAL	Dec. 2012‐2017	NARVAL	Dec. 2012‐2017
CC	26.2	19.1	46.0	26.6
CC_*CT* ≤ 1.3km_	7.9	7.7	12.3	11.5
CC_*CT*>1.3km_	18.6	11.7	35.4	15.8

*Note.* CALIOP = Cloud‐Aerosol Lidar and Infrared Pathfinder; NARVAL = Next‐generation Aircraft Remote‐sensing for VALidation studies campaign. (Top row) total area covered by shallow clouds, with cloud tops below 4 km; (middle row) contributions from clouds with cloud top detected below 1.3 km; and (bottom row) from clouds with cloud top detected above 1.3 km (bottom row). For each cloud category, we provide the mean values over the undisturbed Narval period and the climatological mean over Decembers 2012–2017.

The comparison with LEM‐300 m is actually much better with GridSat, as they both exhibit a daily‐mean total cloud cover of ∼20% and a diurnal amplitude of ∼10%. However, despite this apparently good agreement, GridSat is likely to underestimate the amount of shallow cumulus clouds. This is because it has a wider retrieval footprint as compared to CALIOP and to the ceilometer at BCO, and also because in the infrared channel, shallow cloud tops over warm waters can be more difficult to detect and thus some amount of clouds—presumably the thinnest—might be missed.

### 5.3 Observed Diurnality From Longer‐Term Records

Finally, we examine to what extent this daily cycle can be detected in the observations, on climatological time scales. While still focusing on the boreal winter season, we now consider the full set of measurements which may also include periods with less favorable large‐scale environmental conditions (i.e., near‐surface winds, SST, and subsidence).

Both from the satellite retrievals and in situ measurements at BCO, the climatological daily cycle is noticeable. It shows the same characteristics as during the Narval period, although with a significant reduction in the amplitude in some cases (Figures 8 and 9; Tables 2 and 3). At BCO, the phase of the daily cycle in the ceilometer‐detected cloud covers is preserved (Table 2), which shows that the diurnal variability of shallow cumuli is a robust feature of the winter trades. However, the results in Table 2 and Figure 8e also show that the diurnal amplitude is reduced by a factor of 3 (or more at cloud base), which seems to argue that this daily cycle is subject to some degree of variability, presumably due to a dependence on large‐scale environmental conditions.

The climatological day‐night variations in the total low‐cloud cover from CALIOP is reduced by more than a factor of 2, as compared to the Narval period. This is primarily due to a reduced detection of clouds aloft at nighttime, and secondly during the day (Table 3). The detection of the very shallow clouds is hampered by attenuation of clouds aloft, which leads to a similarly small diurnal cycle amplitude as for the Narval period.

In the GridSat data set, the averaged diurnal amplitude in cloud cover is stronger in DJF (∼8%) than in the December climatology (∼5%; compare the dotted and dash‐dotted lines in Figure 9). Similarly, at BCO, the averaged daily cycle is visible in the DJF climatology (Figure 8), while it is hardly pronounced when only considering December months (not shown). One reason for this might be related to the fact that December is at the transition between the wet and dry season (Brueck et al., 2015; Stevens et al., 2016). Therefore, in some years, the large‐scale environment in December might be less favorable for shallow convection and the overall trade wind conditions that are more typically observed at the heart of the dry season (January, February, and March). Further research is required to understand how the daily cycle of trade wind shallow convection depends on the large‐scale environmental conditions. The point is that depending on the period of analysis and instruments used, it is not always straightforward to detect this daily cycle. This might partly explain why some studies have found a very weak (or no) daily cycle of oceanic shallow convection (Gray & Jacobson, 1977; Kollias & Albrecht, 2010).

## 6 Conclusion and Discussion

The daily cycle of oceanic shallow convection in the trade winds has received little attention in the past. A documentation of this daily cycle is given here in terms of cloudiness and precipitation over the northwest tropical Atlantic ocean upwind of Bardados. The principal findings are based on a 10‐day period with undisturbed conditions in the boreal winter month of December 2013. This period was chosen to take advantage of a set of high‐resolution simulations that have been performed with the ICON model in support of the recent field experiment NARVAL, which took place in December 2013 (Klepp et al., 2014; Stevens et al., 2016, 2019). These simulations are unique in that they are run across a wide range of resolutions (from 2.5‐km to 150‐m grid spacing), over very large domains (up to 9,000 × 3,000 km^2^), and with realistic lateral boundary conditions. In terms of observations, this study also benefits from a large amount of in situ measurements that have been collected at the Barbabos Cloud Observatory (BCO), as well as satellite‐based retrievals that can scan cloudiness over a larger area.

The new investigation tools used here have helped study this daily cycle with a lot more details than was possible 40 years ago when it was first documented (Brill & Albrecht, 1982; Nitta & Esbensen, 1974). The prominent feature of this daily cycle is an evolution of the cloud field between two cloud populations: a population of very shallow cumulus clouds (with tops below 1.3 km), which develops during the day, peaks around sunset, and dissipates throughout the overnight period, and a population of deeper cumuli with stratiform outflow below the trade inversion, which peaks after midnight up to the early morning hours before sunrise. Surface precipitation is strongly connected to the diurnal evolution of the deeper cumuli, with a minimum in the late afternoon and a maximum in the early morning.

This daily cycle is qualitatively consistent among the different model versions and the observations. Longer term statistics from observations also confirm the existence of this daily cycle on climatological time scales. Nevertheless, the detrained layer of stratiform clouds is subject to some uncertainties. More specifically, the model at hectometer‐scale resolutions does not produce enough of these stratiform cloud shields as compared to the observations, while at kilometer‐scale resolutions, it overestimates them. In addition, the nocturnal development of the detrained stratiform clouds aloft tends to be slower in the LEM than in the SRM, leading to an uncertainty in the exact time of the nocturnal peak in total cloud cover. The comparison to the observations indicates that the LEMs best represent this maximum in cloudiness, which occurs at early morning hours before sunrise.

The question now arises as to what mechanisms drive this daily cycle, or what factors can possibly have an influence on it. As discussed in section 3, the SST and associated variations in surface fluxes are likely not to play an important role, as the diurnal variations of the oceanic surface layer are generally weak in the trade winds. No evidence was found either for a role of gravity waves propagating a diurnal signal from nearby tropical lands up to the region of Barbados (not shown). Preliminary analyses, however, suggest the potential importance of radiative effects (not shown), through (i) local changes in stability due to shortwave heating and longwave cooling in the upper boundary layer (Randall & Dazlich, 1991) and (ii) day‐night circulation changes between the cloudy and clear‐sky regions owing to differential radiative cooling/heating rates between cloudy and adjacent clear‐sky areas (Gray & Jacobson, 1977). Further investigation is required to test whether or not these radiative mechanisms are actually dominant. Moreover, the possibility that the daily cycle in trade wind cloudiness be driven by remote influences of diurnally varying deep convection in the intertropical convergence zone is not ruled out (Ruppert, 2016) and will also have to be investigated.

Finally, we want to shed light on the pronounced diurnal variation of cloudiness near cloud base, which essentially arises from nonprecipitating clouds of small vertical extent. This result is robust through all model simulations as well as in measurements at BCO and with CALIOP.

Understanding the factors that control cloudiness near cloud base is of particular interest (e.g., Vial et al., 2016; Vogel et al., 2019), as these clouds are also thought to be critical for the global climate response to warming. This is one of the motivation of the EUREC^4^A (Elucidating the Role of Cloud‐Circulation Coupling in Climate) field campaign (Bony et al., 2017).

## Supporting information



Supporting Information S1

Figure S1

Figure S2

## Appendix A Definition of Cloud Types

### A.1

Figure A1 illustrates the different cloud types and their respective contributions to total cloud cover as defined in this study. The very shallow clouds are defined when cloud top—or more generally, the uppermost cloud side—is lower than 1.3 km (orange). The *clouds aloft* are defined when cloud top (or uppermost cloud side) is above 1.3 km (cyan). The clouds aloft category may be further separated according to the cloud‐base height into a category of *deeper cumuli* (when cloud base is below 1.3 km, violet) and of *inversion clouds* (when cloud base is above 1.3 km, green). The daily cycle of each cloud type is shown in the bottom panel (solid lines). The sum of these three contributions equals the total cloud cover. Because the deeper cumuli alone have a small contribution to total cloud cover and a weak daily cycle, they are considered together with the inversion clouds in one single category of clouds, with cloud top above 1.3 km (cyan curve in Figures A1 and 3).

The definition used to classify clouds in ICON is the same as the one used in CALIOP but is different from that used in BCO. Another category of clouds, with cloud base (or lowermost cloud side) below 1.3 km, is shown in the bottom panel of Figure A1 (orange dash). The definition of this category is the same as that employed with the BCO data (Figures 8d and 8e, orange curve) and in Nuijens et al. (2014, 2015a, 2015b), but with a different height separation, as the cloud‐base cloud fraction peak is slightly higher in the simulations. Note that this cloud category includes both the very shallow clouds and the deeper cumuli. It exhibits a similar daily cycle as the very shallow clouds alone (orange solid), although the period of maximum overcast after sunset tends to last longer (up to midnight). The definition for the inversion clouds (green clouds in Figure A1) is the same as for the BCO data (Figures 8d and 8e, cyan curve), but again with a different height separation.

It is noteworthy that most of cloud‐base detections below 1 km at BCO have a cloud top within the first 1.5 km (in 82% of the cases according to Octave Tessiot's master report). This finding is somewhat consistent with the model results, which indicate that 72% of cloud‐base detections below 1.3 km have also a cloud top below 1.3 km (compare the dashed and solid orange lines in Figure A1). Therefore, the different height separations used in the models and in the observations should have very little, if any, effect on the results.
