# Supplementary material for: A New Look at the Daily Cycle of Trade Wind Cumuli
Source: J Adv Model Earth Syst. 2019 Oct 16;11(10):3148–66. doi: 10.1029/2019MS001746 (PMC6919927; doi:10.1029/2019MS001746)
Supplement: Supplementary file 1 — Supporting Information S1 [file JAME-11-3148-s001.pdf]

Supporting Information for "A new look at the daily cycle of tradewind cumulus clouds"

Jessica Vial[1], Raphaela Vogel[2], Sandrine Bony[2], Bjorn Stevens[1], David M. Winker[3], Xia Cai[4], Cathy Hohenegger[1], Ann Kristin Naumann[1], Hélène Brogniez[5]

[1]Max-Planck-Institut für Meteorologie, Hamburg, Germany

[2]Laboratoire de Météorologie Dynamique, Universite Pierre et Marie Curie (UPMC), Paris, France

[3]NASA Langley Research Center, Hampton, Virginia, USA

[4]Science Systems and Applications, Inc. Hampton, Virginia, USA

[5]Laboratoire Atmosphères, Milieux, Observations Spatiales, Paris, France

Contents of this file

1. Figures S1 to S2

Corresponding author: J. Vial, Laboratoire de Météorologie Dynamique, Universite Pierre et Marie Curie (UPMC), Paris, France (jessica.vial@lmd.jussieu.fr)

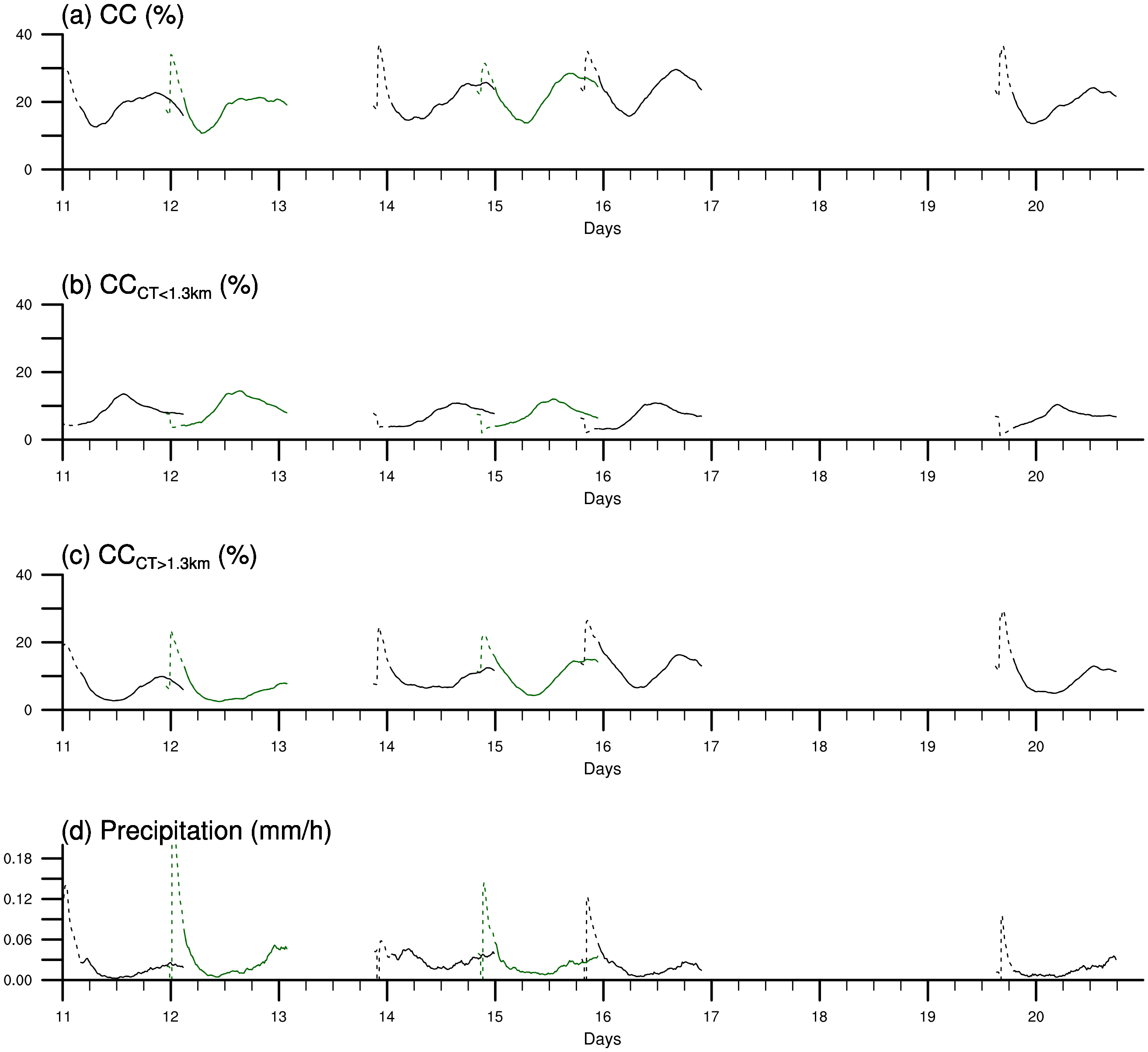

Figure S1. 27-hour time series of total cloud cover (a) and its contributions from very shallow clouds, with tops below 1.3 km (b) and clouds aloft, with tops above 1.3 km (c) and surface precipitation (d) as simulated by LEM-300m for all available days in December 2013. The first four hours of each simulation are considered as spinup and shown in dashed. The alternance between the black and green lines is just to mark the limit between the different days.

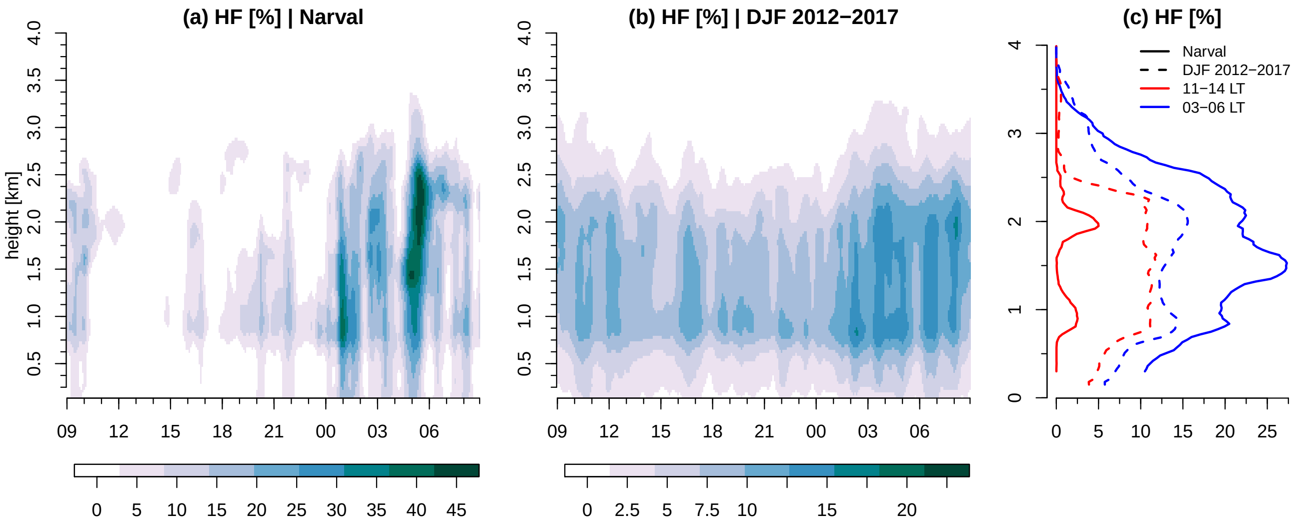

Figure S2. Hydrometeor fraction (HF) for (a) the undisturbed Narval period and (b) the climatology (DJF 2012-2017), and (c) selected profiles for Narval (solid) and climatology (dashed). Note that the data is smoothed with a 1.5-hour centered linear filter, and that the color scale is different for the climatology and for the Narval period (a-b).
