# Supplementary figures and images for: A New Look at the Daily Cycle of Trade Wind Cumuli
Source: J Adv Model Earth Syst. 2019 Oct 16;11(10):3148–66. doi: 10.1029/2019MS001746 (PMC6919927; doi:10.1029/2019MS001746)

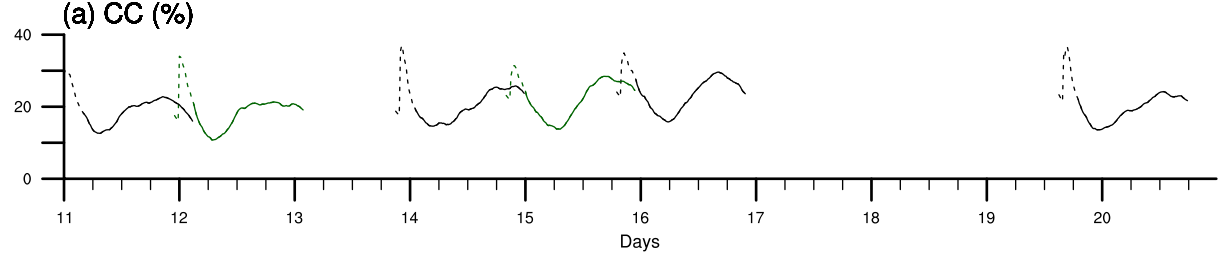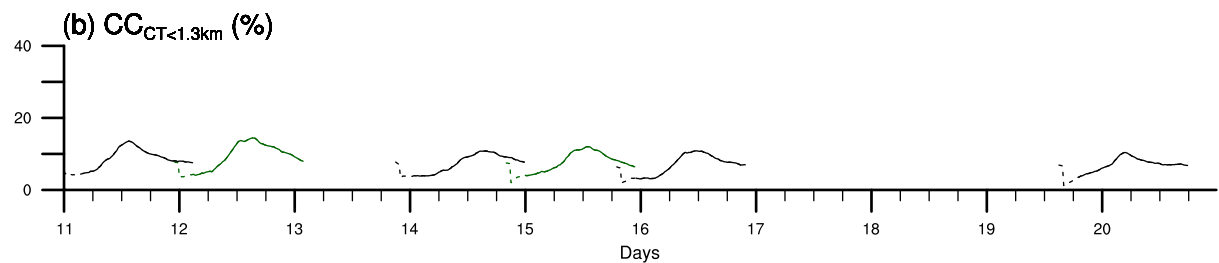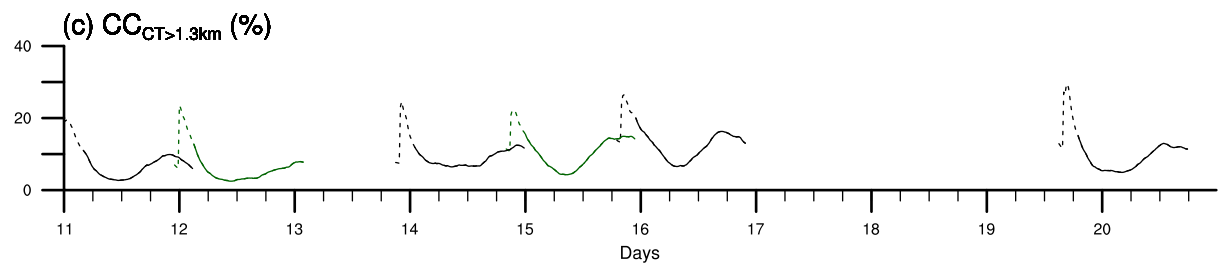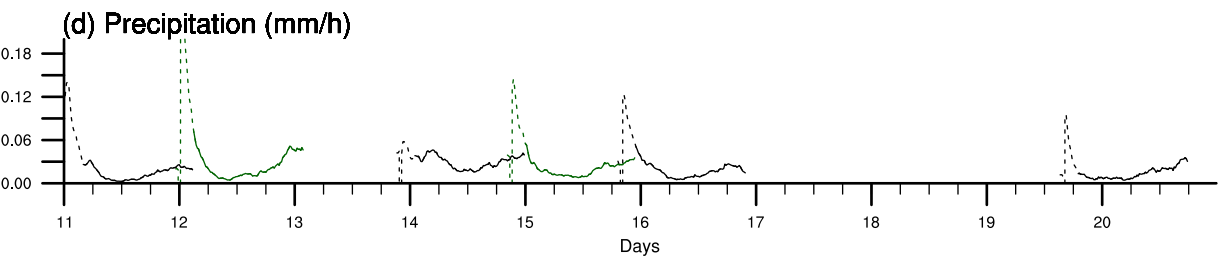

Supplement: Supplementary file 2 — Figure S1 [file JAME-11-3148-s002.pdf]

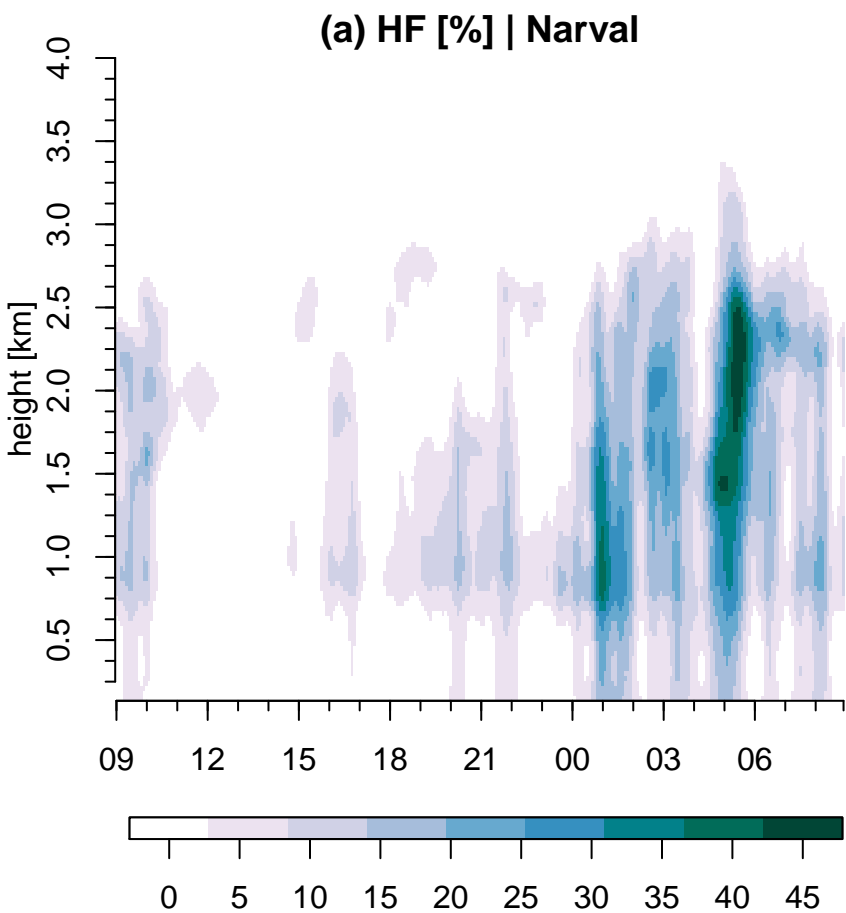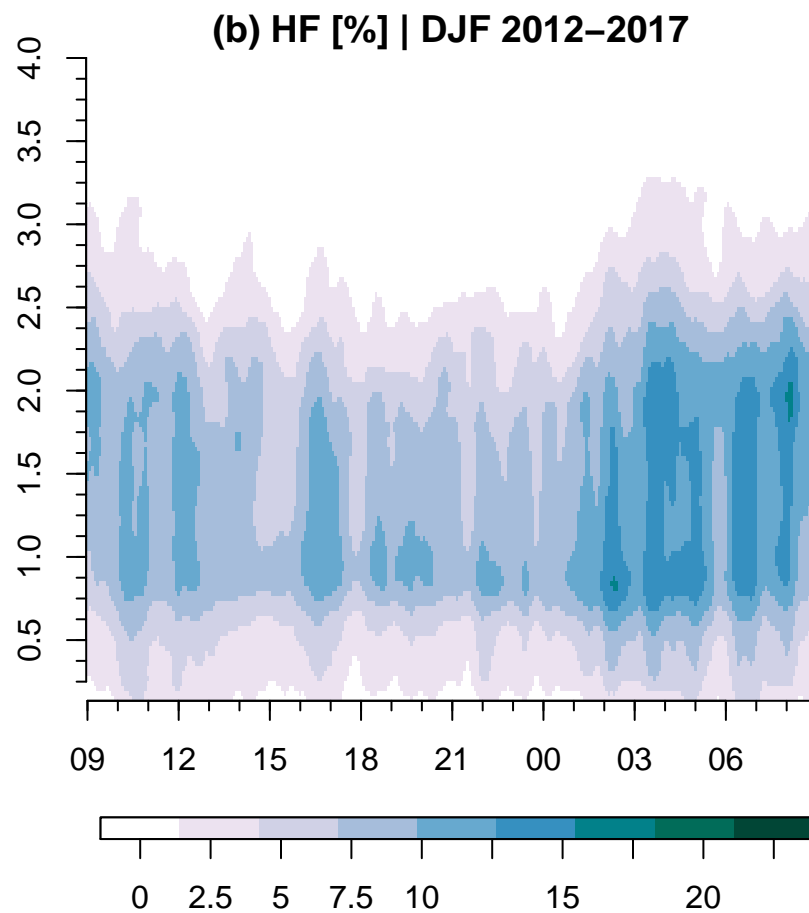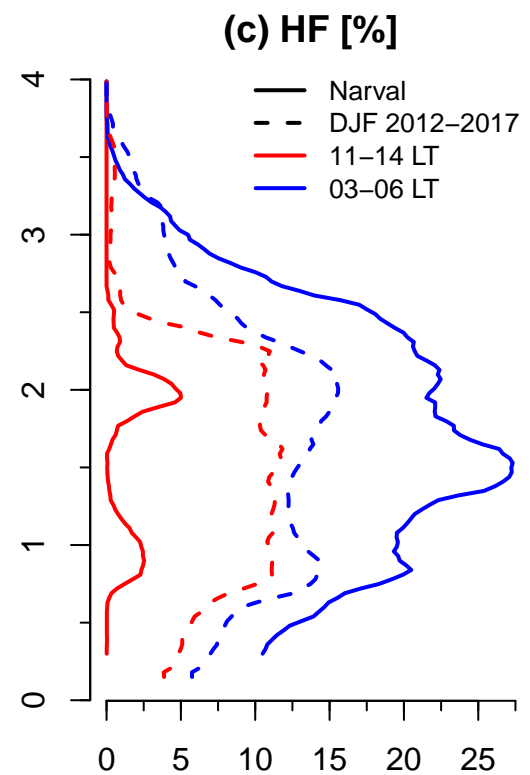

Supplement: Supplementary file 3 — Figure S2 [file JAME-11-3148-s003.pdf]
